# Testosterone and Cortisol Release among Spanish Soccer Fans Watching the 2010 World Cup Final

**DOI:** 10.1371/journal.pone.0034814

**Published:** 2012-04-18

**Authors:** Leander van der Meij, Mercedes Almela, Vanesa Hidalgo, Carolina Villada, Hans IJzerman, Paul A. M. van Lange, Alicia Salvador

**Affiliations:** 1 Laboratory of Social Neuroscience, University of Valencia, Valencia, Spain; 2 Department of Social and Organizational Psychology, VU University Amsterdam, Amsterdam, The Netherlands; CSIC-Univ Miguel Hernandez, Spain

## Abstract

This field study investigated the release of testosterone and cortisol of a vicarious winning experience in Spanish fans watching the finals between Spain and the Netherlands in the 2010 FIFA World Cup Soccer. Spanish fans (*n* = 50) watched the match with friends or family in a public place or at home and also participated in a control condition. Consistent with hypotheses, results revealed that testosterone and cortisol levels were higher when watching the match than on a control day. However, neither testosterone nor cortisol levels increased after the victory of the Spanish team. Moreover, the increase in testosterone secretion was not related to participants' sex, age or soccer fandom, but the increase in total cortisol secretion during the match was higher among men than among women and among fans that were younger. Also, increases in cortisol secretion were greater to the degree that people were a stronger fan of soccer. Level of fandom further appeared to account for the sex effect, but not for the age effect. Generally, the testosterone data from this study are in line with the challenge hypothesis, as testosterone levels of watchers increased to prepare their organism to defend or enhance their social status. The cortisol data from this study are in line with social self-preservation theory, as higher cortisol secretion among young and greater soccer fans suggests that especially they perceived that a negative outcome of the match would threaten their own social esteem.

## Introduction

The finals between Spain and the Netherlands in the 2010 FIFA World Cup for soccer was one of the most important social events of the year. In the Netherlands alone around 8.51 million people (90% of all television viewers) watched the match live on television [1] and in Spain around 15.61 million people (86% of all television viewers) followed the match live on television [2]. Indeed, it beat all records of viewers in television history in both countries.

What does watching such a major event do to people? Obviously, it matters whether one lives in Spain or the Netherlands, and whether one is a fan of soccer or not. But clearly, although people like to think otherwise, soccer outcomes are often not accurately predictable, and part of the excitement may well be rooted in the combination of having a strong preference for who should win, and the basic uncertainty about the very outcome of the game. Especially in this final between the Netherlands and Spain, the outcome was quite uncertain for a large proportion of the match, as the score remained tied during the regular playing time. Only four min before the end of the extra time (116^th^ minute), the Spanish team finally scored the winning goal.

In light of the immense popularity of such an event for many people, it is surprising that so little research had even started to examine the physiological and psychological impact it has on people. The present research seeks to deliver a novel contribution to the literature by examining people's responses to such an important, but uncertain, sport event. We examined not only people's expectations prior to the match and their emotions before and after the match, but also their testosterone and cortisol levels before, during, and after the match. Theoretically, this study extends considerable research on people's responses to winning or losing in a competition, in that we addressed the issue of how vicarious experiences might affect emotional and hormonal responses. Because our focus is on hormonal responses, it is important to address two obvious questions: why testosterone, and why cortisol?

The hormone testosterone is secreted by activation of the hypothalamus-pituitary-gonadal axis and, like cortisol, has many functions in the human body, but of most interest to this study are its functions to maintain or achieve a high social status [3]. In this context, testosterone levels have been shown to increase during competitive events, although these changes are moderated by the appraisal of the situation [4]. From an evolutionary perspective, the challenge hypothesis predicts that testosterone levels increase in challenging contexts that are relevant for reproduction [5], [6]. These challenging contexts also include competition if the outcome of the competition would increase the social status of the winner. In support of this idea, a high motivation to win has been positively related to changes in testosterone levels during competition [7], and an increase in testosterone levels has been observed when competitors are confronted with a challenging opponent [8]. However, the effect of winning or losing on testosterone levels is unclear, as some studies have related increases in testosterone levels to the experience of winning [9]–[11], but others have failed to do so [7], [8], [12], [13].

Up until now, only one other study has investigated changes in testosterone levels among soccer fans watching a World Cup final soccer match [14]. During the final match of the 1994 FIFA World Cup between Brazil and Italy, the authors found an increase in testosterone levels among fans of the winning team and a decrease in testosterone levels among fans of the losing team. In our study, we argue that the social status of Spanish fans was at stake, since the social status of the group to which they belonged would certainly be affected by either losing or winning. Therefore, we expected an elevation in testosterone levels among Spanish fans during the match.

The hormone cortisol is the end product of the activation of the hypothalamus-pituitary-adrenal axis and has many functions in the human organism, but of most interest to this study is its stress-regulatory functions related to competitive encounters [15]. Many studies have shown that a wide range of stressful events produce increases in cortisol levels, such as jumping out of an airplane [16] or performing a public speaking task in front of an audience [17], [18]. Increases in cortisol levels during such events are thought to improve performance in the short-term by, for example, increasing the available amount of energy through an increase in glucose levels, whereas in the long-term, high cortisol levels stop the stress response and revert the organism to homeostasis (for a review see [19]). Along these lines, cortisol levels have been shown to increase before the start of a competition [20], and that cortisol levels increase during competition in a wide variety of sports, such as soccer [21], judo [7], and rowing [22]. These changes may partly be explained by mere physical effort, since exercise is a stressor in itself [23]. Yet, cortisol levels have also been shown to increase in competitions that do not require exerting any physical effort, such as Japanese chess [24].

The increase in cortisol secretion in the context of a competition can be explained by social self-preservation theory, which predicts that cortisol levels increase in contexts where social status or acceptance is threatened [25]. Evidence for this theory is provided by studies showing that cortisol levels increase in situations where one's self-identity can be negatively judged by others [26] and when the outcome of a negative situation is beyond one's control [27]. Clearly, a match played by one's national sports team fits both these characteristics; as losing may result in a clear threat to one's identity, often through challenging if not derogatory comments from fans from rival teams, while fans cannot directly control the outcome of a match. Consequently, as in the case of testosterone, we expected an elevation in cortisol levels among Spanish fans throughout the match.

The goal of this field study was to investigate cortisol and testosterone secretion in male and female Spanish fans of different ages watching the final soccer match of the 2010 FIFA World Cup. These fans also participated in a control condition, mainly to control for anticipatory effects on hormonal levels. We also investigated whether these hormonal changes were different according to the sex and age of the fans, and we explored whether hormonal changes were influenced by soccer fandom, expectations before the match, and situational appraisal after the match.

## Materials and Methods

### Participants

In total, 58 persons participated in this study; however, we excluded 8 participants because they had a medical condition or used drugs that influence cortisol and/or testosterone levels and/or influence the experience of emotional situations [28]. The specific causes for exclusion were: pregnancy (1), depression (2), use of corticosteroids (1), use of anxiolytic medication (2) and daily marihuana use (2). This left us with a final sample size of 50 participants: 25 men (Mean = 37.8 years ± s.e.m. = 2.8) and 25 women (34.0±2.2). Including these 8 excluded participants in our analysis did not change the statistical conclusions of the main results.

Among the women who participated, 8 were using hormonal contraception, 13 were not using hormonal contraception and 4 were postmenopausal. The sample had the following average socio-demographic characteristics: 35.9 years old (±1.8), body-mass index of 23.6 (±0.4), sported per week 1:06 h (±0∶16), drank 3.9 alcohol units per week (±0.8), smoked 2.5 cigarettes per day (±0.7), and slept per day 7:09 h (±0∶08). Average self-perceived socio-economic status (1 = lowest through 10 = highest) was 6.1 (±0.2) [29]. Furthermore, out of all the participants, 80% were in a relationship, 32% were parents, 32% were students, 70% had full-time jobs, 36% practised a sport, and 30% smoked.

Up to 1 hour before the match and during the match, participants were asked not to smoke or eat. However, several participants did not comply with these instructions since 15 participants smoked one or more cigarettes (5.0 cigarettes ±0.8), 7 participants did eat a sandwich or something similar, and 15 participants drank alcohol (3.3 alcohol units ±0.6). To control for these confounds, the participants' consumption and the time of consumption were monitored and written down by the experimenter, and in the control condition they were instructed to consume the same amount at the same time as they had during the match.

All participants received basic verbal information about the study and signed an informed consent form outlining the general procedure and the measurements taken. This study was approved by the ethical committee of the University of Valencia and conformed to the Declaration of Helsinki.

### Procedure

This study used a cross-over design with an experimental condition (day of the match) and a control condition (days after final: 16.0±2.2). Participants watched the final soccer match of the 2010 FIFA World Cup in different groups, with each group led by an experimenter. The experimenters were the authors of this study and colleagues of the authors at the University of Valencia. Each experimenter received thorough instructions about how to coordinate the session several days before the match. During the match, 18 participants were in the company of a group of only friends (no. of persons in group: 6.6±0.8), 18 participants were in the company of friends and family (no. of persons in group: 17.7±1.1), 13 participants were in the company of only family (no. of persons in group: 2.9±0.3). Participants watched the match in a public place (*n* = 11) or at home (*n* = 39).

This particular soccer match was composed of a first half (45 min), a half-time break (15 min), a second half (45 min), and two periods of 15 min extra time (30 min). After regular time (the first two halves), the score was 0-0, which seemed to produce a lot of tension among fans. Four min before the end of the extra time (116^th^ minute), the Spanish team scored the winning goal. Just before the match, participants completed a questionnaire measuring their expectations and mood while they provided a saliva sample (S1). During the half-time break, they provided a second saliva sample (S2). At the end of the match, they filled in a questionnaire about how they perceived the match and a mood questionnaire, while providing the last saliva sample (S3).

In the control condition, participants were instructed to be with the same persons and in the same location as during the match, but without any exciting stimuli (e.g., no parties or watching an exciting movie). At the same times as during the match, participants filled in the same mood questionnaires and provided the three saliva samples. Additionally, participants filled in a general health and habit questionnaire. Five participants refused to take part in the control condition.

### Questionnaires

#### Fandom

Before the start of the match participants answered questions measuring to what extent they perceived themselves as soccer fans. We measured this by standardising (z-scores) and averaging the following questions: (i) *How much of a fan are you of the Spanish national soccer team during this world championship?*, (ii) *How much of a fan are you of the Spanish national soccer team in other matches it plays?*, (iii) *How much of a fan are you of another soccer team?*, (iv) *How much do you like soccer?*, and (v) *How many soccer matches do you watch per month?* (Cronbach's alpha: 0.83). Apart from the last question, which had an open answer, participants answered the questions on a 7-point Likert scale (1 = not at all to 7 = extremely).

#### Expectation

Before the match, we asked participants if they thought Spain or the Netherlands was going to win and we asked them what end score they expected. We also measured perceived importance of the match by asking (on a scale from 1 to 100): *How important is it to you for Spain to win the final?*

#### Situational appraisal

After the match, participants completed questions regarding their perception of the match (modified from [8]). We measured the perceived difficulty/effort required for the Spanish team by averaging the following two questions: (i) *How much effort did the match require from the Spanish team?* and (ii) *How difficult was the match for the Spanish team?* (Cronbach's alpha: 0.67). We also asked the participants how well they thought the Spanish team had played. Furthermore, they were also asked about how frustrating and stressful watching the match was for them (e.g., *How frustrating/stressful was watching the final for you?*). Participants answered each question on a 7-point Likert scale (1 = not at all, 7 = extremely).

#### Mood

We measured the participants' positive and negative mood before and after the match and at the corresponding times on the control day, by using the PANAS questionnaire, translated into Spanish, and subsequently validated [30]. The scale consisted of ten items describing positive mood (e.g. enthusiastic, activated) and ten describing negative mood (e.g. ashamed, irritable). For each item, participants were required to indicate the extent to which it corresponded with their current mood (1 = not at all, 5 = very much). Across conditions, for positive feelings we found an average Cronbach's alpha of 0.87 (±0.02), and for negative feelings the figure was 0.89 (±0.06). Positive and negative mood scores were created by taking the sum of their items.

### Biochemical analysis

In each condition, three saliva samples were collected by means of passive drooling. Participants deposited 5 ml of saliva in plastic vials which took approximately 10 minutes to fill. In the experimental condition, the first saliva sample (S1) was provided just before the start of the match (CET: 20∶15), the second saliva sample (S2) was provided during the half-time break (CET: 21∶20), and the third saliva sample was provided 20 min after the end of the match (CET: 23∶15), which was 24 min after the winning goal. The time difference between S1 and S2 was 65 min, between S2 and S3 115 min, and between S1 and S3 3 hours. The sampling times in the control condition were the same as in the experimental condition. Biochemical analyses were conducted by the Laboratory of Social Neuroscience at the University of Valencia, Spain.

Salivary cortisol levels were determined in duplicate with the Spectria Cortisol RIA kit (cat. n° 06119) from Orion Diagnostica (Espoo, Finland). The detection limit of this kit was 0.8 nmol/l, and the mean inter- and intra-assay coefficients of variation were all below 8%.

Salivary testosterone was determined in duplicate using enzyme-immunoassays with the expanded range salivary testosterone enzyme-immunoassay kit (cat. n° 1-2402) from Salimetrics (Suffolk, UK). The detection limit of this kit was <1.0 pg/ml, and the mean inter- and intra-assay coefficients of variation were all below 10%.

Excluding participants who had in one or more of their saliva samples a hormonal concentration that deviated by more than three standard deviations from the mean did not change the statistical conclusions of the main results. However, it did change the *p* value of the post hoc tests of the factor Fandom in the match to marginal significance (*p = *0.082).

### Statistical analysis

We first investigated with independent *t*-tests whether men and women differed in their soccer fandom, expectancies before the match, and their situational appraisal after the match had ended.

We used linear mixed modeling to investigate changes in mood and hormonal levels before and during the match and the control condition. As an estimation method we used the restricted maximum likelihood procedure since this procedure deals better with outliers [31]. To allow for differences in patterns between participants, we included a random component for the six moments and a random component for each subject. To analyze hormonal levels, we added the following factors: (i) Moment (1 = CET 20∶15; 2 = CET 21∶20; 3 = CET 23∶15), (ii) Condition (0 = control; 1 = match), (iii) Sex (1 = man, 2 = woman), and as a covariate (iv) Age (standardized). When we investigated mood we used the previous model, with the exception that the factor Moment had only two levels (pre and post) and that the random component had four levels. We log transformed age and the testosterone and cortisol values because they were positively skewed.

We started with the most complex model with all possible interactions and then progressively removed non-significant effects, starting with the most complex effects. For illustrative purposes, we always maintained the interaction between Moment and Condition. After removal of a factor we investigated whether this improved model fit according to the criteria of Akaike's Information Criterion (AIC) and Schwarz's Bayesian Information Criterion (BIC). See in Appendix S1 for the results of these analyses for mood (see Appendix S1, Table 1 and 2), cortisol (see Appendix S1, Table 3) and testosterone (see Appendix S1, Table 4). For the calculation of AIC and BIC we used the maximum likelihood procedure in SPSS because it gives more reliable estimates than the restricted maximum likelihood procedure. We considered a lower value of at least 2 in one or both criterions as a better model [32].

To investigate if the psychological factors reported in paragraph 2.4 influenced cortisol and testosterone secretion we added in separate steps each factor (standardized) and its interaction with Condition in the previously constructed models of cortisol and testosterone (see Table 5 in Appendix S1 for the change in model fit for each factor we added). We investigated mediation by bootstrapping [33].

For post hoc tests we used the correction of Sidak. A value of *p*<0.05 (two-tailed) was considered statistically significant. Statistical tests were performed with SPSS version 17.0. Values are mean ± s.e.m. when not otherwise specified. For illustrative purposes, we used raw scores for the calculation of the percentage change and effect sizes and for the values in the figures.

## Results

### Fandom, expectation and situational appraisal

Before the match, all participants thought Spain was going to win on average with a 1.44 goal difference (±0.10) and thought it was important to win the match (61.64±4.73 on a scale from 1 to 100). Men and women did not differ in how difficult they perceived the match for the Spanish team, nor did they differ in how important they perceived the match or how stressful it was for them watching the match (all *p*≥0.193). However, men did expect a bigger goal difference than women (men: 1.64±0.16; women: 1.24±0.10, *t*_48_ = 2.07, *p* = 0.043), and although marginally significant, men found the match more frustrating (men: 3.16±0.34; women; 2.16±0.41, *t*_48_ = −1.88, *p* = 0.067) and were bigger soccer fans than women (men: 0.22±0.16; women: −0.21±0.14, *t*_48_ = 2.00, *p* = 0.051).

### Mood response

#### Positive mood

The model predicting positive mood showed that there was a significant interaction between Condition and Moment (*F*_1,105.08_ = 12.02, *p* = 0.001). This change is illustrated by the fact that positive mood increased from pre match to post match on average by 14% (*t*_24.41_ = 4.84, *p*≤0.001, Cohen's *d* = 0.54), while it did not change during the control condition (*t*_36.17_ = −0.97, *p* = 0.339). There was also a main effect of Condition (*F*_1,112.76_ = 176.12, *p*≤0.001), showing that participants reported on average a 50% higher positive mood on the day of the match than on the day of the control condition (Cohen's *d* = 1.60). Adding other main and interaction effects to the model did not improve model fit (see Table 1 in Appendix S1).

#### Negative mood

The model predicting negative mood showed that there was a main effect of Condition (*F*_1,125.59_ = 21.28, *p*≤0.001). Participants reported on average a 20% higher negative mood on the day of the match than on the day of the control condition (Cohen's *d* = 0.58). There was also a significant interaction between Sex and Age, showing that, independent of the two conditions, in women, older age was related to less negative mood (*β* = −0.329, *t*_39.33_ = −2.21, *p* = 0.033), whereas age was not related to negative mood among men (*β* = 0.118, *t*_39.11_ = −0.90, *p* = 0.375). Condition did not interact with Moment (*F*_1, 115.44_ = 0.05, *p* = 0.820), and model fit did not improve when adding other main effects (e.g. Sex and Age) or interaction effects (see Table 2 in Appendix S1).

### Hormonal response

#### Testosterone

**Figure 1:**
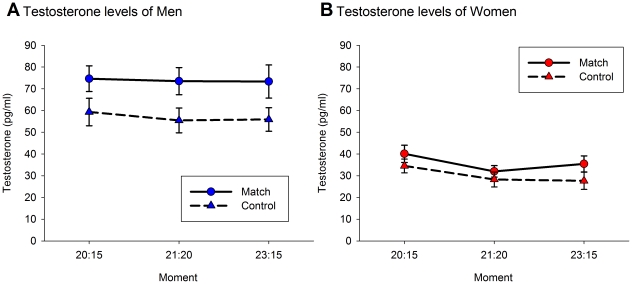
Mean (± s.e.m.) testosterone levels at 20∶15, 21∶20 and 23∶15 (CET) during the match and control day depicted for male (1A) and female (1B) fans.

The model predicting testosterone levels showed that there was a main effect of Condition (*F*_1, 223.32_ = 23.23, *p*≤0.001). Testosterone levels were on average 29% higher during the match than on the control day (Cohen's *d* = 0.52, see Fig. 1). Condition did not interact with Moment (*F*_2, 127.08_ = 0.57, *p* = 0.566). There was a main effect of Sex, showing that men had overall higher testosterone levels than women (*F*_1, 47.17_ = 50.05, *p*≤0.001). Model fit did not improve when adding other main effects or interaction effects (see Table 3 in Appendix S1).

#### Cortisol

**Figure 2:**
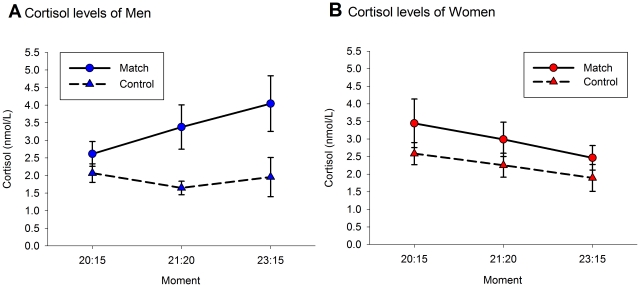
Mean (± s.e.m.) cortisol levels at 20∶15, 21∶20 and 23∶15 (CET) during the match and control day depicted for male (2A) and female (2B) fans.

The model predicting cortisol levels showed that there was a main effect of Condition (*F*_1,207.92_ = 24.63, *p*≤0.001). Cortisol levels were on average 52% higher during the match than on the control day (Cohen's *d* = 0.83, see Fig. 2). There was also an interaction between Sex and Condition (*F*_1, 195.71_ = 4.72, *p* = 0.031). Men secreted 77% more cortisol on the day of the match than on the day of the control condition (t_229.36_ = 4.95, *p*≤0.001, Cohen's *d* = 1.16), and women secreted 32% more cortisol on the day of the match than on the day of the control condition (*t*_214.15_ = 2.11, *p* = 0.036, Cohen's *d* = 0.53). Additionally, there was an interaction between Age and Condition (*F*_1, 197.57_ = 6.86, *p* = 0.010), showing that older participants secreted less cortisol on the day of the match (*β* = −0.250, *t*_72.01_ = −2.26, *p* = 0.027), whereas age was not related to cortisol secretion on to the day of the control condition, (*β* = −0.018, *t*_56.46_ = −0.18, *p* = 0.858). Finally, there was a marginally significant interaction between Moment and Condition (*F*_2, 124.83_ = 2.74, *p* = 0.068). Results showed that, compared to baseline, cortisol levels in the control condition were to a marginally significant extend lower in the second sample (*t*_36.20_ = 2.27, *p* = 0.085, Cohen's *d* = 0.27), and significantly lower in the third sample (*t*_56.73_ = 3.39, *p* = 0.004, Cohen's *d* = 0.30). However, cortisol levels did not change during the match (all *p*≥0.900). Model fit did not improve when adding other interaction effects (see Table 4 in Appendix S1).

### The influence of psychological factors on hormonal levels

#### Testosterone

The addition of Fandom, Expectations and Situational Appraisal to the model predicting testosterone levels did not further improve model fit (see Table 5 in Appendix S1).

#### Cortisol

**Figure 3:**
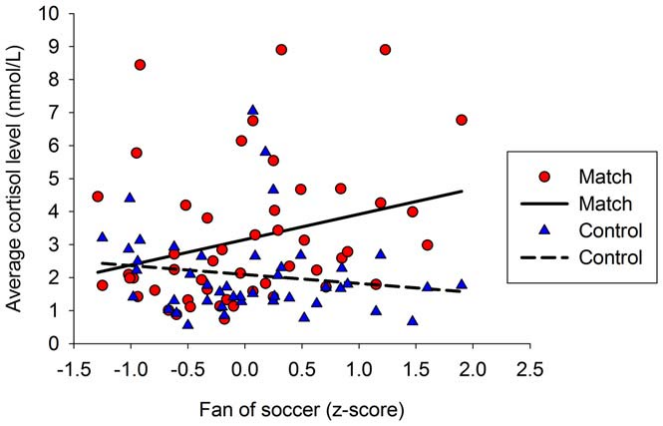
The relationship between fandom and the total cortisol secretion during the match and the control day.

Only the addition of Fandom to the model predicting cortisol levels further improved model fit, whereas the addition of Expectations and Situational Appraisal did not improve model fit (see Table 5 in Appendix S1). In this model, there was an interaction between Fandom and Condition (*F*_1, 202.85_ = 12.50, *p* = 0.001). Fandom appeared to mediate the previous interaction between Sex and Condition since due to the inclusion of Fandom this interaction was no longer significant. The mediation of Fandom was supported by bootstrapping (95% CI: [−0.60, −0.06]). Including Fandom in the model also changed the interaction between Age and Condition to marginal significance, but Fandom did not mediate the effect of Age on Condition (95% CI: [−1.31, 0.08]). Participant's Fandom was positively related to a larger cortisol secretion on the day of the match (*β* = 0.225, *t*_70.03_ = 2.04, *p* = 0.045), whereas on the day of the control condition Fandom had no influence on cortisol secretion (*β* = −0.134, *t*_56.90_ = −1.32, *p* = 0.193, see Fig. 3).

## Discussion

This field study investigated hormonal changes in response to a vicarious experience among Spanish fans watching the final soccer match of the 2010 FIFA World Cup. The main findings of the study were that both testosterone and cortisol concentrations among Spanish fans were elevated while watching the match as compared to the control day. That the match was engaging to Spanish fans was not only shown by elevated testosterone and cortisol levels during the match, but also by an elevated positive mood and high expectations.

Testosterone levels were higher during the match than during the control condition and are therefore in line with the challenge hypothesis [5], [6]. According to this hypothesis, testosterone levels should increase during contexts that are challenging and relevant for the social status of the winner. The World Cup final can certainly be viewed as challenging since it was uncertain who would win the match and the outcome would influence the social status of the group to whom the fans belonged. As a result, testosterone levels of fans probably increased to prepare them to defend or enhance their social status. However, we did not find a winner effect, since there was no increase in testosterone levels at the end of the match when the Spanish team had won. This finding contradicts a similar study by Bernhardt et al. [14], who found an increase in testosterone levels among fans of the winning team of the 1994 World Cup final match. However, the latter finding needs to be interpreted with some caution, because their article did not include any post-hoc significance tests investigating whether testosterone levels increased among the fans of the winning team. Furthermore, the sample size was limited (12 Brazilian fans and 9 Italian fans), there was no control condition, and there was no information about many other relevant factors, such as participants' health and participants' consumption during the match. Nevertheless, an important difference between our study and the study of Bernhardt et al. [14] is that the Brazilian and Italian soccer fans watched the game in the US, whereas in the present study the fans watched the game in their home-country. It could therefore be argued that the lack of testosterone increase in our study could be related to a lack of the presence of a ‘competing’ out group since in our study fans would encounter few or no fans with a different affiliation. However, the different findings between our study and the one from Bernhardt et al. [14] may well reflect the general tendency in the literature that testosterone levels and a winning experience do not always “go hand in hand” [4], [15]. Instead, it has been proposed that changes in testosterone levels as a reaction to competitive situations are mediated by psychological processes, such as performance appraisal and causal attribution [15].

The elevated cortisol secretion during the match can be explained by the social self-preservation theory [25]. According to this theory, the hypothalamus-pituitary-adrenal axis gets activated in reaction to a threat to the social-self, and consequently, cortisol is released. We think the World Cup final soccer match posed such a threat, as for many fans their social status depended on the performance of their national team. This was even more true because this was no ordinary soccer match, as the soccer status of their nation was at stake, the match was broadcast all over the world, and the result of the match was completely uncontrollable for the Spanish fans.

Watching the match was probably more stressful for men than for women as male fans secreted more cortisol during the match than female fans. Indeed, male fans expected a bigger goal difference, and although marginally significant, reported to be bigger soccer fans and perceived the match as more frustrating than women. However, it appeared that the sex of fans was not the most import predictor of cortisol levels as the sex difference disappeared when predicting cortisol levels with the fan's self-reported soccer fandom. Irrespective of fans' sex, especially big soccer fans experienced greater cortisol secretion during the match. This last finding actually agrees with the social self-preservation theory, as the social status of people who particularly support the team should be connected to a greater degree to the outcome of the match. An increase in cortisol levels during such competitive encounters is thought to be an adaptive response, since it improves performance during these specific encounters by, for example, increasing glucose levels [19]. Thus, in the specific situation of watching the final soccer match, an increase in cortisol levels among fans could be an adaptive response, since it would prepare them to face and cope with negative reactions from their environment in the case of losing the match.

Why did younger fans secrete more cortisol on the day of the match than older fans? According to the stress literature, the reverse pattern is usually observed, showing that older people secrete more cortisol in response to physiological, psychological, or pharmacological challenge (for a meta-analysis see [34]). This greater cortisol response has usually been explained as a progressive loss in negative feedback sensitivity of the hypothalamus-pituitary-adrenal axis with older age [35]. We think that this discrepancy between our results and those from the stress literature can be explained by the type of stressor used to evoke an increase in cortisol release. Life experience may not act as much as a protector against stress-evoking situations in the laboratory, but it may be a benefit in more real-life stress-evoking situations such as watching one's nation's team play the final match of a world soccer cup. In this particular real-life situation, older fans may have appraised and coped with the stressor better than younger fans (i.e. they were less stressed), thereby experiencing less total cortisol secretion. This reasoning is supported by researchers arguing that emotional and physiological reactions to stressors are mediated by appraisal of the stressor and coping behaviour [36]. Underscoring this, it has been shown that older people tend to use coping strategies indicative of greater impulse control and the tendency to positively appraise conflict situations, whereas younger adults use strategies that are outwardly aggressive and psychologically undifferentiated, indicating lower levels of impulse control and self-awareness [37].

The results from this field study illustrate the importance of individual differences when studying hormonal changes in competitive contexts. Among Spanish fans watching the final soccer match of the 2010 FIFA World Cup, we found that their cortisol responses were influenced by their sex, their age, and their soccer fandom. Before closing, we wish to note that the present findings give rise to an interesting scientific puzzle. The results revealed that before the onset of the match *all* Spanish watchers expected the Spanish national team to win from the Dutch national team. Such level of consensus is quite exceptional, but seems to suggest that the Spanish were quite confident about the outcome of the match. With this collectively shared optimism in mind, it was interesting to see that the cortisol and testosterone levels were considerably elevated during the match. One might speculate that expressing a belief in winning might serve to some degree as a mechanism to cope with uncertainty and the risk of losing. After all, the outcomes of soccer finals are often unpredictable. At the very least, the present findings suggest important discrepancies between hormonal data, which suggest increases in stress and risk for social self-esteem, and the expressed expectations regarding the outcome of the match, which suggests a minor increase in stress and risk. Perhaps in such exceptional circumstances, where the psychological stakes are high, outcomes are not really predictable, and beyond one's control, we cannot always completely count on what people say. This suggests the importance of future research to uncover the specific features of the situations in which cognitive and physiological responses seem to be at odds or not.

## Supporting Information

Appendix S1 **Supplemental Tables.** Table 1: Fit of the various models predicting positive mood. Table 2: Fit of the various models predicting negative mood. Table 3: Fit of the various models predicting testosterone levels. Table 4: Fit of the various models predicting cortisol levels. Table 5: Change in model fit for the models predicting cortisol and testosterone when adding psychological factors. Original model fit testosterone: AIC = −184.38, BIC = −133.24. Original model fit cortisol: AIC = 67.65, BIC = 129.74.
(DOC)
