# Supplementary material for: Testosterone and Cortisol Release among Spanish Soccer Fans Watching the 2010 World Cup Final
Source: PLoS One. 2012 Apr 18;7(4):e34814. doi: 10.1371/journal.pone.0034814 (PMC3329546; doi:10.1371/journal.pone.0034814)
Supplement: Appendix S1 — Supplemental Tables. Table 1: Fit of the various models predicting positive mood. Table 2: Fit of the various models predicting negative mood. Table 3: Fit of the various models predicting testosterone levels. Table 4: Fit of the various models predicting cortisol levels. Table 5: Change in model fit for the models predicting cortisol and testosterone when adding psychological factors. Original model fit testosterone: AIC = −184.38, BIC = −133.24. Original model fit cortisol: AIC = 67.65, BIC = 129.74. (DOC) [file pone.0034814.s001.doc]

**Appendix S1**

**Table 1:** Fit of the various models predicting positive mood.

| **Model** | **Removed variable** | **AIC** | **BIC** |
| --- | --- | --- | --- |
| Complete model | None | 388.23 | 455.75 |
| Removal of ns. 4-way interaction | All | 386.25 | 450.55 |
| Removal of ns. 3-way interactions | All | 379.15 | 430.59 |
| Removal of ns. 2-way interactions | All except Condition×Moment | 374.08 | 409.45 |
| Removal of ns. main effects | Age Sex | 371.37 | 400.30 |
|  |  |  |  |

**Table 2:** Fit of the various models predicting negative mood.

| **Model** | **Removed variable** | **AIC** | **BIC** |
| --- | --- | --- | --- |
| Complete model | None | 253.08 | 320.59 |
| Removal of ns. 4-way interaction | All | 252.06 | 316.35 |
| Removal of ns. 3-way interactions | All | 247.49 | 298.92 |
| Removal of ns. 2-way interactions | Moment×Age Moment×Sex Condition×Sex Condition×Age | 243.39 | 281.97 |
| Removal of ns. main effects | None | - | - |
|  |  |  |  |

**Table 3:** Fit of the various models predicting testosterone levels.

| **Model** | **Removed variable** | **AIC** | **BIC** |
| --- | --- | --- | --- |
| Complete model | None | -168.60 | -55.37 |
| Removal of ns. 4-way interaction | All | -171.08 | -65.16 |
| Removal of ns. 3-way interactions | All | -179.84 | -99.48 |
| Removal of ns. 2-way interactions | All except Condition×Moment | -184.63 | -129.84 |
| Removal of ns. main effects | Age | -184.38 | -133.24 |
|  |  |  |  |

**Table 4:** Fit of the various models predicting cortisol levels.

| **Model** | **Removed variable** | **AIC** | **BIC** |
| --- | --- | --- | --- |
| Complete model | None | 81.15 | 194.37 |
| Removal of ns. 4-way interaction | All | 77.35 | 183.27 |
| Removal of ns. 3-way interactions | All | 70.13 | 150.49 |
| Removal of ns. 2-way interactions | Moment×Age Moment×Sex Sex×Age | 67.65 | 129.74 |
| Removal of ns. main effects | None | - | - |

**Table 5:** Change in model fit for the models predicting cortisol and testosterone when adding psychological factors. Original model fit testosterone: AIC = -184.38, BIC = -133.24. Original model fit cortisol: AIC = 67.65, BIC = 129.74.

|  | **Testosterone** | | **Cortisol** | |
| --- | --- | --- | --- | --- |
| **Factor added** | **ΔAIC** | **ΔBIC** | **ΔAIC** | **ΔBIC** |
| Soccer Fandom | 0.84 | 8.14 | -7.69 | 0.38 |
| Importance | 1.17 | 8.47 | -1.85 | 5.45 |
| Difficulty/effort Spain | -0.78 | 6.52 | 3.34 | 10.64 |
| Performance Spain | -4.36[[1]](#footnote-2) | 2.77 | -0.63 | 6.48 |
| Goal Difference | 2.53 | 9.83 | 3.47 | 1078 |
| Frustrating | 0.35 | 7.65 | 3.81 | 11.12 |
| Stressful | 3.22 | 10.52 | 3.19 | 10.50 |
|  |  |  |  |  |

1. This factor only improved model fit according to AIC and worsened model fit according to BIC. In this model, there was only a main effect of perceived Spanish performance and no interaction with Condition and thus this factor shall not be discussed. [↑](#footnote-ref-2)
